# Machine learning of correlated dihedral potentials for atomistic molecular force fields

**DOI:** 10.1038/s41598-018-21070-0

**Published:** 2018-02-07

**Authors:** Pascal Friederich, Manuel Konrad, Timo Strunk, Wolfgang Wenzel

**Affiliations:** 1 Institute of Nanotechnology, Karlsruhe Institute of Technology, Hermann-von-Helmholtz-Platz 1, 76344 Eggenstein-Leopoldshafen, Germany; 2 Nanomatch GmbH, Hermann-von-Helmholtz-Platz 1, 76344 Eggenstein-Leopoldshafen, Germany

## Abstract

Computer simulation increasingly complements experimental efforts to describe nanoscale structure formation. Molecular mechanics simulations and related computational methods fundamentally rely on the accuracy of classical atomistic force fields for the evaluation of inter- and intramolecular energies. One indispensable component of such force fields, in particular for large organic molecules, is the accuracy of molecule-specific dihedral potentials which are the key determinants of molecular flexibility. We show in this work that non-local correlations of dihedral potentials play a decisive role in the description of the total molecular energy—an effect which is neglected in most state-of-the-art dihedral force fields. We furthermore present an efficient machine learning approach to compute intramolecular conformational energies. We demonstrate with the example of α-NPD, a molecule frequently used in organic electronics, that this approach outperforms traditional force fields by decreasing the mean absolute deviations by one order of magnitude to values smaller than 0.37 kcal/mol (16.0 meV) per dihedral angle.

## Introduction

Molecular dynamics (MD) methods are widely used for the simulation of inorganic and organic materials at the atomistic level, for example in the field of computational biology and drug design but also in the field of organic electronics for organic light emitting diodes, organic solar cells and other technologically relevant applications. Computational material characterization and development of organic semiconductors is based in large parts on force field based methods to simulate the structure of molecular materials during thin film formation. Such atomistic simulations help to gain insight into experimentally accessible microscopic processes and mechanisms. Force field based approaches can furthermore be combined with other simulation techniques such as density functional theory or continuum simulations to scale bridging simulation workflows^1–3^. Such multiscale modeling techniques can be used to generate a fully operational digital twin of real devices. This digital pendant can be analyzed and optimized to reduce costly experiments and to finally gain better insight into device functionality and the interplay of processes on different length and time scales^4–6^. High accuracy in each of the multiscale simulation steps is required to generate reliable and predicitve models.

MD simulations use classical force fields to describe the interaction between atoms and molecules. Essentially all state-of-the-art force fields, such as the OPLS, AMBER and GROMOS force fields consist of terms which describe bonded and non-bonded interactions of molecular materials^7,8^. The bonded interactions model intramolecular potentials and typically consist of bond length contributions, angle contributions and dihedral angle contributions. The latter are parameterized using linear combinations of periodic functions of individual dihedral angles *ϕ*:

1 $$\begin{array}{c}{E}_{{\rm{dihedrals}}}=\sum _{{\rm{dihedrals}}}(\frac{{V}_{1}}{2}(1+cos(\varphi -{\varphi }_{1})+\frac{{V}_{2}}{2}(1-cos(2\varphi -{\varphi }_{2})\\ \quad \quad \quad +\frac{{V}_{3}}{2}(1+cos(3\varphi -{\varphi }_{3})+\frac{{V}_{4}}{2}(1-cos(4\varphi -{\varphi }_{4}))\end{array}$$

*V*_*i*_ and *ϕ*_*i*_ are dihedral specific force field parameters obtained from *ab initio* calculations or empirical studies. These models work with sufficient accuracy for specific classes of materials, while other materials, especially organic molecules consisting of several flexibly bound aromatic ligands and side groups, require molecule-specific dihedral potentials^9^. These are parameterized using scans of the intramolecular energy of a molecule during independent rotation of each dihedral angle. The potential energies are then either used to parameterize models as shown in Equation (1) or are directly tabulated and used during the force field evaluation. Recognizing the problem of missing correlation terms there have been efforts to define and parameterize correlated force fields, such as the class 2 force field^10^ COMPASS^11^, which includes correlation terms between bond lengths and dihedral angles. These correlations terms drastically increase the number of free force field parameters making generic as well as molecule-specific parameterization costly and difficult.

**Figure 1:**
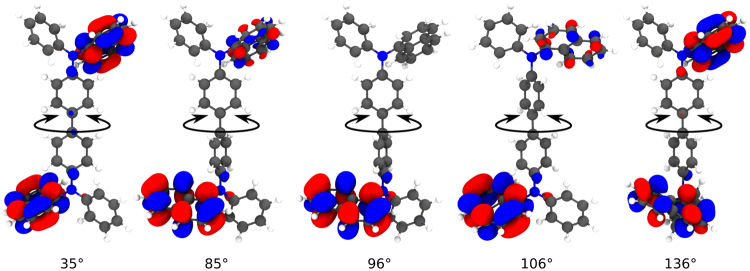
Lowest unoccupied molecular orbital (LUMO) of α-NPD at different configurations of the central dihedral angle. The localization of the orbital significantly changes at approximately orthogonal ring planes.

We show here that for many molecules an accurate description of the total energy requires correlated force fields which concurrently consider several dihedral angles. Consequently, the standard approach to model the energy as a sum of terms depending only on one dihedral angle leads to significant deviations between the energy as determined by electronic structure methods and the force field energy. These deviations result in part from branched molecular structures and delocalized electronic states (see Fig. 1) which result in correlations of quantum mechanical origin between dihedral angles that are difficult to capture in state-of-the-art force fields. These effects are particularly important for large organic molecules with bulky ligands, such as those used in applications of organic electronics.

We furthermore show in this paper that the abovementioned problem of missing correlations in standard force fields can be solved using machine learning models which accurately predict internal molecular energies of random dihedral conformations of molecules. As a proof of principle we discuss how this approach can directly be used in our Metropolis Monte Carlo based simulated annealing protocol^12,13^ for the generation of large amorphous morphologies, which only requires energy evaluations. We finally discuss straightforward extensions of the artificial neural network approach for gradient prediction which is necessary for the application in widely used MD models.

## Uncorrelated dihedral force fields

**Figure 2:**
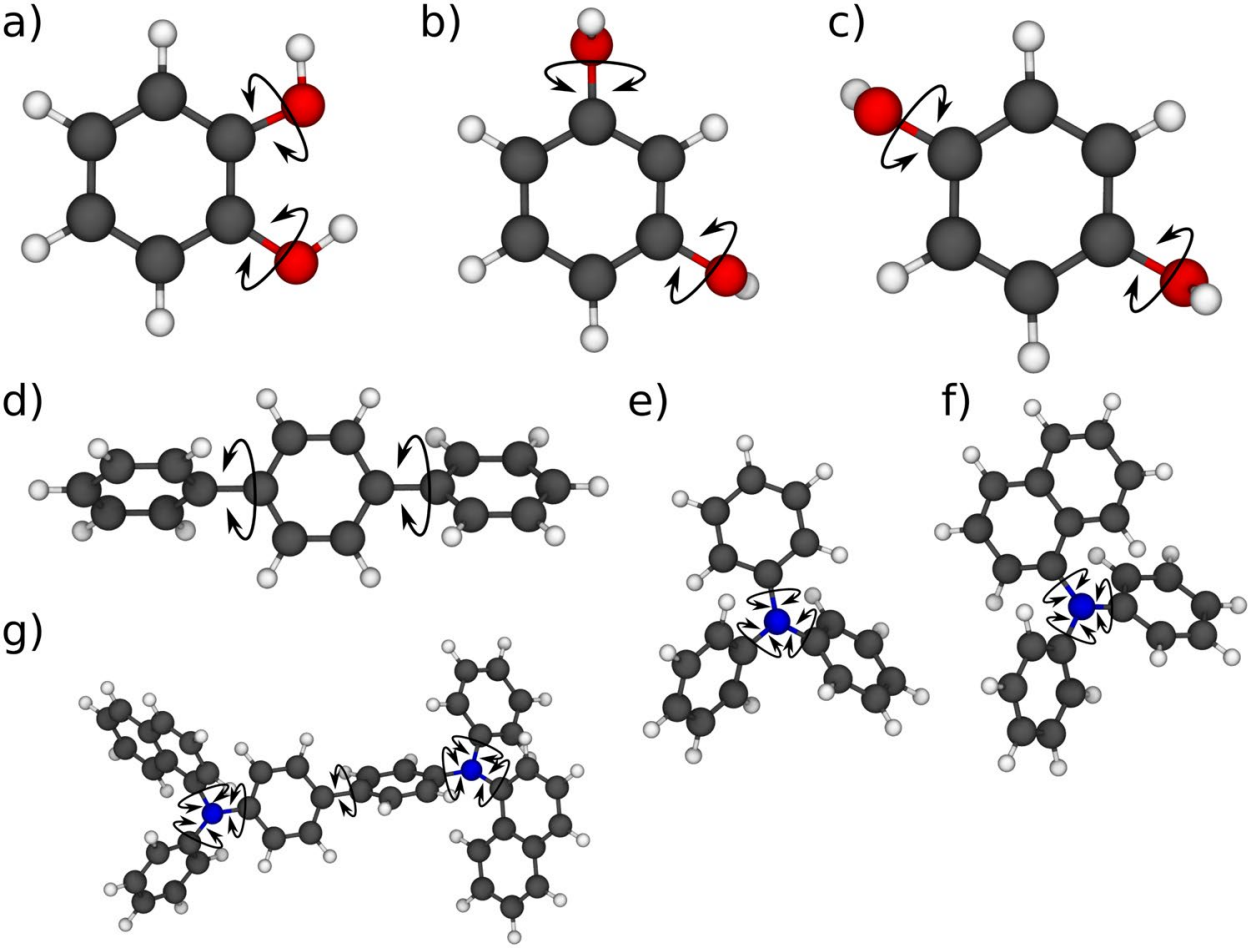
Molecules used as test cases for the dihedral energy calculation: (**a**) BD12, (**b**) BD13, (**c**) BD14, (**d**) DPB, (**e**) TPA, (**f**) DPNA and (**g**) α-NPD.

To investigate the accuracy of different dihedral force fields, we parameterize molecule-specific force fields for a set of molecules (see Fig. 2) using semi-empirical PM7 calculations as implemented in MOPAC 2016^14,15^. The selected molecules comprise of three or more rigid subunits connected by dihedral angles in a linear or branched way. During parameterization, we keep bond lengths and angles within the molecular subunits constant to avoid mixing of dihedral potentials with contributions coming from variation of bond lengths and angles.

**Figure 3:**
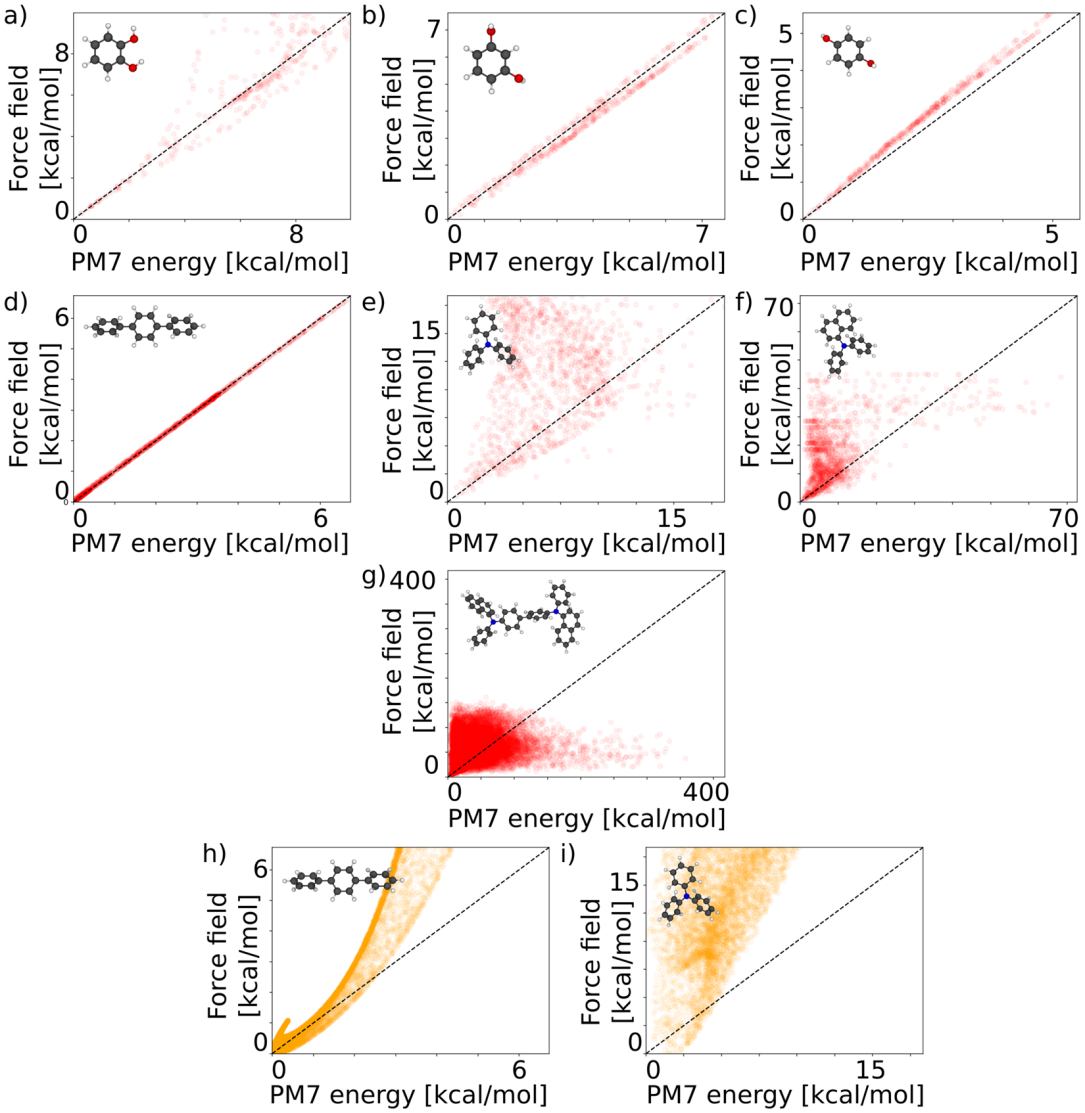
Correlation between the internal energy of random configurations of the molecules shown in Fig. 2 evaluated using a non-correlated force field approach and using the semi-empirical PM7 method. Panels a–g show the results of a molecule-specific dihedrals-only force field, while panels h-i show the energies predicted by the standardized GROMOS 54A7 force field^21^ parameterized using the Automatic Topology Builder^22^.

After parameterization of the molecule-specific uncorrelated force field, we generate random dihedral configurations and compare the force field energies of these configurations to their respective PM7 energies. Figure 3 shows the comparison of intramolecular force field energies and PM7 energies of benzene-1,4-diol (BD12), benzene-1,4-diol (BD13), benzene-1,4-diol (BD14), 1,4-Diphenylbenzol (DPB), triphenylamine (TPA), N-diphenyl 1-naphthylamine (DPNA) and N,N′-Di(1-naphthyl)-N,N′-diphenyl-(1,1′-biphenyl)−4,4′-diamine (α-NPD). We find good agreement between the uncorrelated force field energy and the PM7 energy for the linear BD compounds and the linear TP, while for the branched amine based compounds TPA, DPNA and α-NPD the correlation is weak (pearson product moment correlation of −0.069, 0.492 and 0.181, respectively). For the benzene-diol compounds the force field accuracy increases with increasing distance of the hydroxyl groups. For branched aromatic molecules, such as TPA/DPNA and more complex derivatives such as α-NPD the force field approach fails to predict the internal molecular energy, indicating that the total energy of the molecule cannot be written as additive terms of the dihedral energies. This can be attributed in part to the fact that for branched molecules with a particular dihedral conformation the electronic energy is a complex function of the internal degrees of freedom.

These data suggest that consideration of correlations between dihedral angles may improve the deviations of the uncorrelated force field approach and the PM7 energies. The method which is presently used for the parameterization of uncorrelated dihedral potentials independently samples the rotation of each of the *n*_d_ dihedral angles using *n*_s_ sampling points. It thus requires *n*_s_·*n*_d_ energy evaluations for the parameterization. A fully correlated model which systematically samples the entire conformational space requires ${({n}_{{\rm{s}}})}^{{n}_{{\rm{d}}}}$ energy evaluations, which is computationally infeasible for realistic molecules with 5–10 dihedral angles and sampling steps of *e.g*. 5° (*n*_s_ = 72).

One possible solution to this problem are the use of machine learning algorithms with the ability to learn the optimal single angle dihedral potentials as well as their correlations. In the following sections we investigate an artificial neural network which is trained using random dihedral configurations of a molecule to predict the molecular energy of all possible configurations.

## Training of the artificial neural network

For the prediction of correlated dihedral energies, we use artificial neural networks with one input layer, two or three hidden layers and one output layer. All hidden layers consist of logistic neurons with an activation function

2 $${h}_{j}^{i}({x}_{j}^{i})=\frac{1}{1+\exp (-{x}_{j}^{i})}$$

where *i* denotes the layer of the neuron, *j* its position within the layer (see Figure S1) and ${x}_{j}^{i}={\vec{b}}_{j}^{i-1}+{({{\boldsymbol{\omega }}}^{i-1}{\vec{h}}^{i-1})}_{j}$ is the input of the activation function, evaluated using the output of the previous layer (*i* − 1) and the weight matrices ***ω***^*i*^ and the biases *b*^*i*^, which are optimized during the training.

The input vector includes the values *α*_*i*_ of the *n*_d_ dihedral angles in a given molecular conformation as well as sine and cosine values of these angles (sin(*kα*_*i*_), cos(*kα*_*i*_)) with periodicities *k* in 0.5, 1.0, 2.0, …, 10.0. Training was performed using the stochastic gradient based Adam method^16^ and a mini batch size of 200. 72% of the samples were used for the training of the network. We used L2 regularization and early stopping with a validation set size of 13% of the input samples to overcome problems of overfitting. The remaining 15% of the input samples were used to test the training efficiency and to validate the force field. We furthermore used different numbers and sizes of hidden layers and used model averaging^17^ to improve the accuracy of the results. In particular, we averaged over predictions by the five artificial neural networks shown in Table 1. For training of the artificial neural network, we use the implementation in the python scikit-learn package^18^.

**Table 1:** Parameters of the five artificial neural networks used for the prediction of intramolecular energies of molecules with random dihedral configurations.

	Number of hidden units		
	Hidden layer 1	Hidden layer 2	Hidden layer 3
Network 1	30	10	—
Network 2	100	10	—
Network 3	110	7	—
Network 4	60	30	10
Network 5	50	20	8

## Correlated dihedral force fields

The input conformations for the training of the artificial neural network were generated using random sets of dihedral angles. Each conformation was checked for clashes which were defined as non-bonded atom distances below 2.1 Å. Conformations with clashes were disregarded and replaced by valid random conformations. The total energies of conformations were calculated using the semi-empirical PM7 method and shifted by the energy of the optimized dihedral conformation with the globally lowest energy.

We tested the method on the seven molecules shown in Fig. 2 including, as the most complex example, α-NPD, a widely used hole transport material for applications in organic light emitting diodes^4,19,20^. For the α-NPD molecule, we stochastically generated 150,000 dihedral configurations and calculated the energies of these configurations. We then trained and evaluated the artificial neural network as described in the last section using these 150,000 input samples. 108,000 samples (72%) were used for training, 19500 samples (13%) were used for validation (early stopping) and 22500 samples (15%) were used as test cases. We furthermore calculated the conformational energies using an uncorrelated force field which we parameterized using independent energy scans of all dihedral angles (see also Fig. 2). For the BD materials, we used 3000 random dihedral configurations while for DPB, TPA and DPNA we used 10000 random conformers. The percentages used for training, validation and testing were the same for all materials.

**Figure 4:**
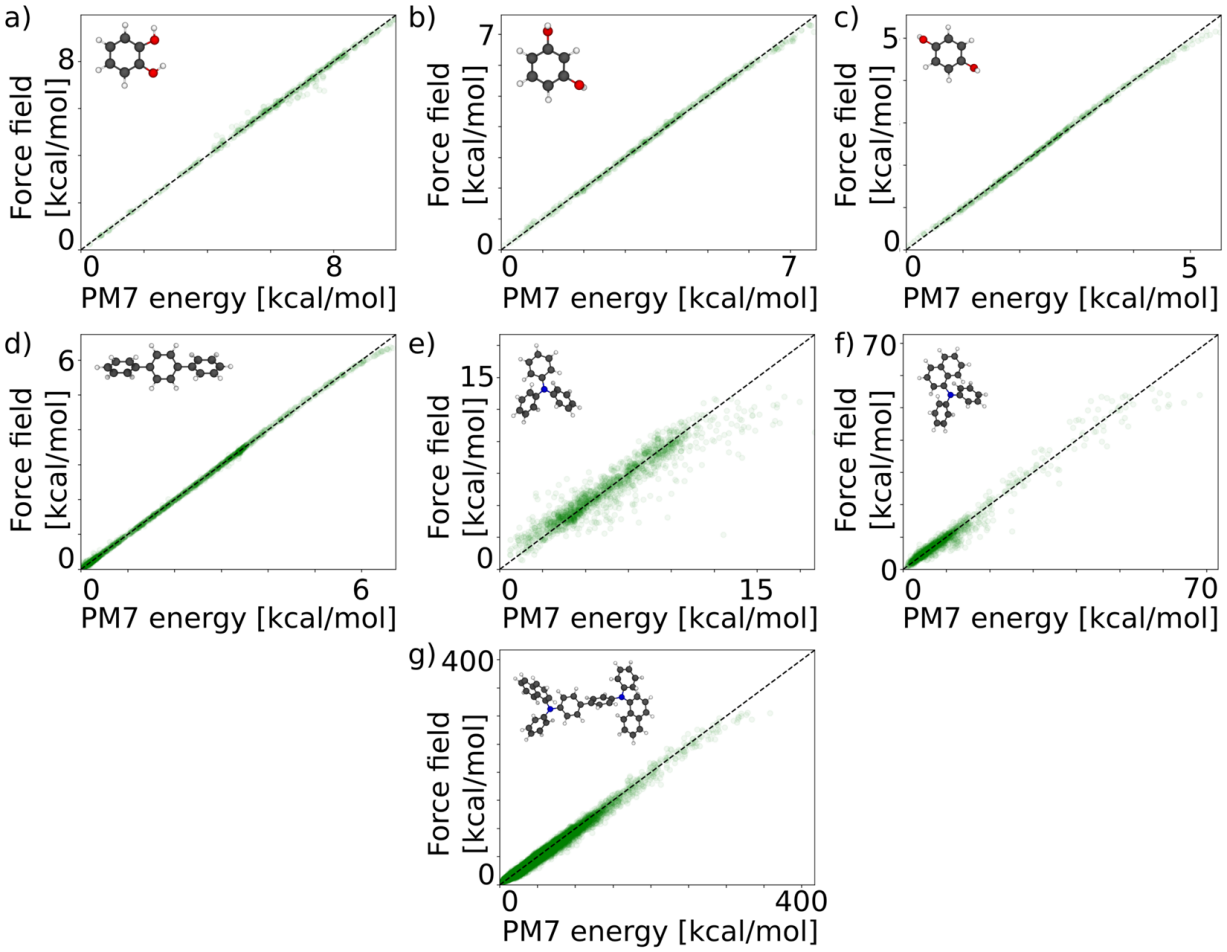
Correlation between the internal energy of random configurations of the molecules shown in Fig. 2 evaluated using the artificial neural network and using the semi-empirical PM7 method.

**Figure 5:**
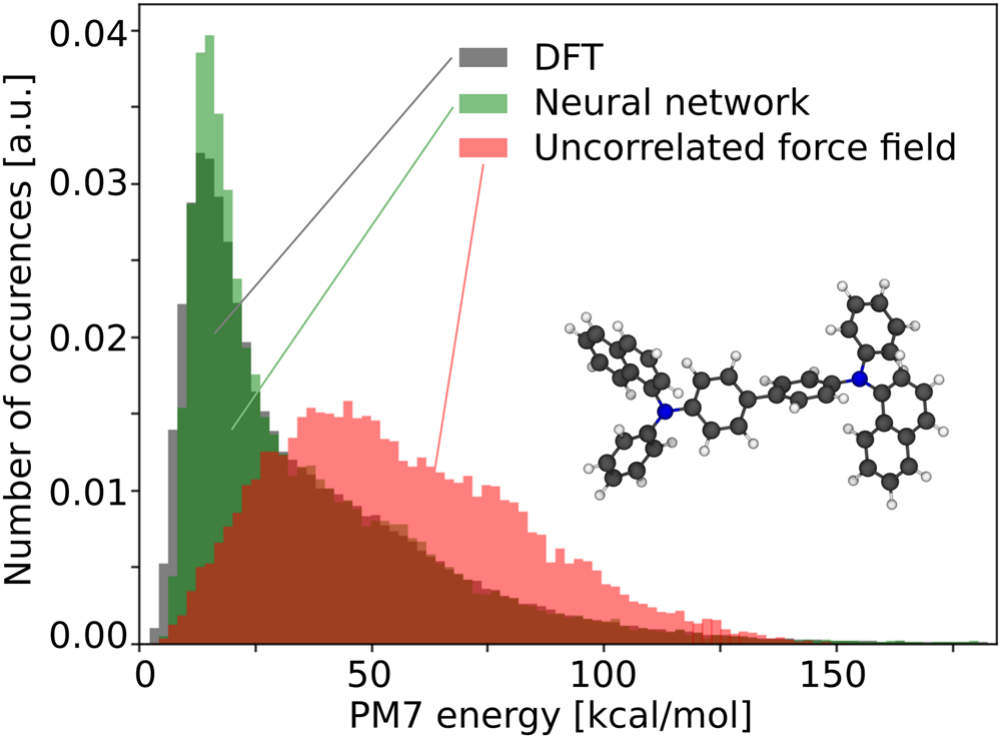
Histogram of conformational energies of α-NPD calculated using PM7, the artificial neural network and an uncorrelated conventional force field model.

The results in Fig. 3 show that the dihedral-angle-only force fields using uncorrelated terms struggle to accurately reproduce the PM7 energies. Also the standard GROMOS 54A7 force field^21–29^ which, apart from dihedral potentials also contains explicit Lennard-Jones and internal electrostatic terms which in principle lead to correlations between the dihedral angles, fails to predict the PM7 energies of DPB and TPA (see Fig. 3h,i). The artificial neural network approach (Fig. 4) on the other hand accurately predicts the PM7 energies of 22500 random dihedral configurations which were not used in the training of the net. In case of α-NPD, the accuracy (mean absolute deviation) of the artificial neural network is 0.37 kcal/mol (16.0 meV) per dihedral angle, while the uncorrelated model (Fig. 2) shows a mean deviation of 4.63 kcal/mol (200.7 meV) per dihedral angle. For conformations with energies smaller than 11.5 kcal/mol (0.5 eV) the accuracy of the artificial neural network approach increases and the mean deviation reduces to 0.29 kcal/mol (12.6 meV) per dihedral angle. The Pearson product-moment correlation coefficients for TPA, DPNA and α-NPD are 0.944, 0.984 and 0.995, respectively. The deviation of the uncorrelated model mainly arises from overestimation of the conformational energies, which is reflected in a shift of the peak position in the energy histogram in Fig. 5. The distribution of energies predicted by the artificial neural network nicely matches the energy distribution of the target energies. Once the artificial neural network is trained, the energy evaluation is a simple series of matrix-vector multiplications which can easily be parallelized for efficient force field evaluation. The application in a force field based Monte Carlo approach as well as further extensions of the model will be discussed in the next section. An analysis of the artificial neural network coefficients is shown in Figure S2.

## Low energy classification

Efficient application of the artificial neural network approach in force field based simulations requires a fast computation of the internal molecular energy and thus a fast evaluation of the artificial neural network. The dihedral force field has to be evaluated in each simulation step which requires an efficient implementation of the forward pass which is basically a series of matrix vector multiplications. In Monte Carlo based approaches as described in Neumann *et al*.^12,13^, and Friederich *et al*.^30,31^, each Monte Carlo steps consists of a random change in the system followed by the evaluation of the Metropolis Monte Carlo criterion which compares the system before and after each random move. The probability *p* of accepting moves which increase the energy by Δ*E* drops exponentially as *p* ∝ *exp*(−Δ*E*/(*k*_*B*_*T*)). During the simulation of a molecular system at a given temperature *T*, intermolecular alignment reduces the energy of the total system while the internal energy of single molecules increases as a trade-off. It is therefore sufficient to accurately predict conformational energies which are equal to typical van der Waals bonds between molecules. Conformational energies of more than 23–46 kcal/mol (1–2 eV) are unlikely to occur at typical simulation temperatures between 300 K and 400 K.

**Figure 6:**
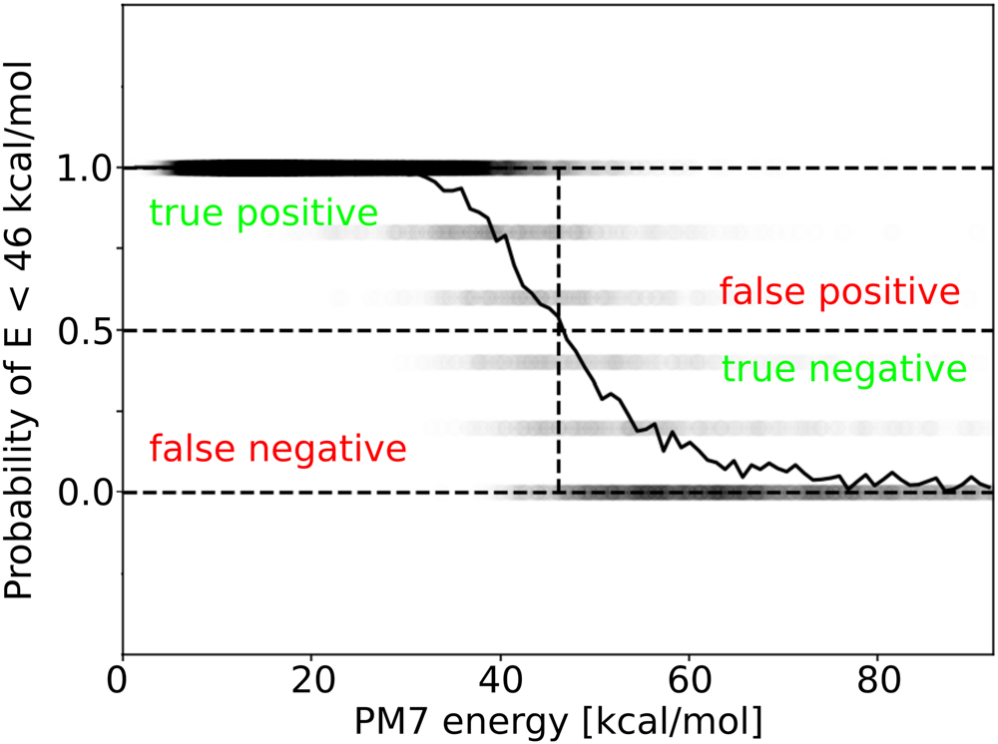
Prediction of the classification network with the task to identify conformations with energies smaller than 46 kcal/mol (2 eV). The solid line shows a moving average of the predictions shown as black dots. The confusion matrix is shown in Table 3.

To avoid the artificial neural network based energy calculation for high energy conformations occurring during the random moves of a Monte Carlo algorithm, a fast classifier to distinguish between low and high energy conformations is needed. We therefore implemented a fast classification network to separate low from high energy conformations. The much larger artificial neural network for energy *prediction* will then only be used in case the faster *classification* network recognizes a low energy conformation. The classification network was trained using threshold energy of 46 kcal/mol (2 eV) on the α-NPD input data used in the last section. We again use five different artificial neural network parameters (see Table 2) and average the results of all 5 networks, leading to classification probabilities 0.0, 0.2, 0.4, 0.6, 0.8 and 1.0. The results are shown in in Fig. 6. The averaged solid line shows that most of the dihedral configurations with energies smaller than 46 kcal/mol (2.0 eV) are recognized as low energy conformations. The confusion matrix which measures the number of true positive (TP), false positive (FP), false negative (FN) and true negative (TN) samples is given in Table 3, with a sensitivity of the prediction of TP/P = 96.5%, a specificity of TN/N = 88.3%, an accuracy of (TP + TN)/(TP + TN + FP + FN) = 94.2% and a F1 score of 2TP/(2TP + FP + FN) = 96.0%. The network furthermore detects conformations with energies smaller than 23 kcal/mol (1 eV) with a probability of 100%. The strongly reduced size of the artificial neural network used for classification compared to the neural network for energy prediction (compare Tables 2 and 3) makes energy classification much faster compared to neural network based energy prediction. By avoiding many costly but unnecessary evaluations of high energy conformations, the combination of both neural networks will speed up the computation time in a Monte Carlo based protocol for morphology simulation.

**Table 2:** Parameters of the five artificial neural networks used for the classification of intramolecular energies of molecules with random dihedral configurations.

	Number of hidden units	
	Hidden layer 1	Hidden layer 2
Network 1	30	—
Network 2	40	—
Network 3	50	—
Network 4	5	5
Network 5	10	3

**Table 3:** Confusion matrix of the classification results shown in Fig. 6.

True positive: 15655	False positive: 738
False negative: 560	True negative: 5547

## Conclusions

In this work, we demonstrated that an artificial neural network can serve for energy of the intramolecular degrees of freedom of complex organic molecules. In principle, this approach can be extended to gradients, by either taking the derivative of the network energy function or training additional networks. We have not pursued this here because energy gradients are not used in the Monte Carlo method which we use to generate the morphologies of the components in organic devices^2,6^. When the model is trained to predict a vector of torsion gradients, Cartesian gradients for each atom are easily obtained by transformation of the internal gradients which only requires the moments of inertia of the rigid molecular subunits.

The method presented here can furthermore be used to parameterize soft degrees of freedom such as dihedral rotations in coarse grained force fields. Here, comparably rigid molecular subgroups are represented by single pseudo-atoms connected by flexible or rotatable bonds. Dihedral rotations are in many cases the only intramolecular degrees of freedom taken into account in coarse grained representations of molecules, making an accurate parameterization of these degrees of freedom even more relevant. The missing substructure of the pseudo-atoms of coarse grained models and thus missing explicit intramolecular Lennard Jones interaction between atoms pose additional challenges to coarse grained force fields which are at least partially solved in the approach presented in this study.

We have shown in this investigation that a multi-layer artificial neural network can efficiently and accurately capture the correlations of dihedral angles in complex organic molecules and generate an accurate potential for all dihedral angles, while conventional uncorrelated force fields struggle to give proper estimates of the conformational energy. The artificial neural network is trained on random conformations and reproduces the conformational energies with high accuracy. This method can directly be applied in force field based Monte Carlo simulations of small molecules, as used for the simulation of *e.g*. organic electronic devices such as organic light emitting diodes^12^. It can furthermore be extended to calculate energy gradients and forces for atomistic molecular mechanics simulations.

For future applications, it may be possible to extend this approach to train intramolecular force fields for larger molecular structures such as polymers and proteins for which highly accurate force fields are required for reliable structure prediction. To achieve this generalization of the method, the moderately local nature of the correlated torsion angles can be exploited.

### Availability of materials and data

The force field parameterization and the parameters of the artificial neural networks generated during the current study are available from the corresponding author on reasonable request.

## Electronic supplementary material

Supplementary Information
