# Supplementary material for: Machine learning of correlated dihedral potentials for atomistic molecular force fields
Source: Sci Rep. 2018 Feb 7;8:2559. doi: 10.1038/s41598-018-21070-0 (PMC5803249; doi:10.1038/s41598-018-21070-0)
Supplement: Supplementary file 1 — Supplementary Information [file 41598_2018_21070_MOESM1_ESM.pdf]

Supplementary Information

Machine learning of correlated dihedral potentials for atomistic molecular force fields

*Pascal Friederich, Manuel Konrad, Timo Strunk, Wolfgang Wenzel**

Topology of the neural network

The topology of the artificial neural network with two hidden layers is shown in **Figure S1**. It consists of one input layer, two hidden layers and one output layer, interconnected by the weight matrices $\boldsymbol{\omega}^i$ and the bias vectors $\vec{b}^i$.

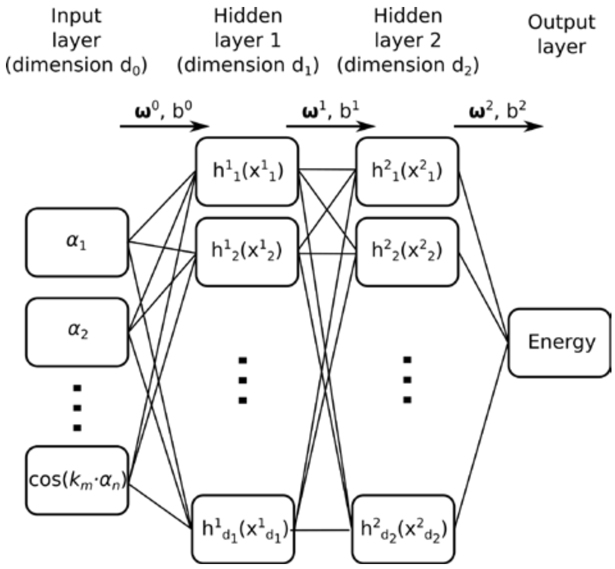

Figure S1. Topology of a artificial neural network with two hidden layers. The input layer includes all dihedral angles α_i as well as $\cos(k_j\alpha_i)$ and $\sin(k_j\alpha_i)$ terms for all periodicities k_j given in the main text.

Analysis of the weights ω^1 connecting the input and the first hidden layer

Figure S2 shows for each input channel of the artificial neural network the sum of the absolute values of the coefficients connecting the input channel to the first hidden layer ("integrated intensity"). These sums can serve as a rough estimate of the influence of each input channel to the final energy prediction. The angles α_1 to α_7 (see **Figure S3**) in the first seven channels have only weak connections to the first hidden layer which might indicate that there are only small linear components in the torsion potentials. The $\cos(k\alpha)$ and $\sin(k\alpha)$ input channels have stronger weights with small to medium periodicities k between 0.5 and 5.0 being more important than high frequencies up to 10.0. The dihedral angle between the two central phenyl rings of α-NPD shows significantly different weights than the other dihedral angles which indicates that it is more independent from the other angles, which are likely to be more correlated.

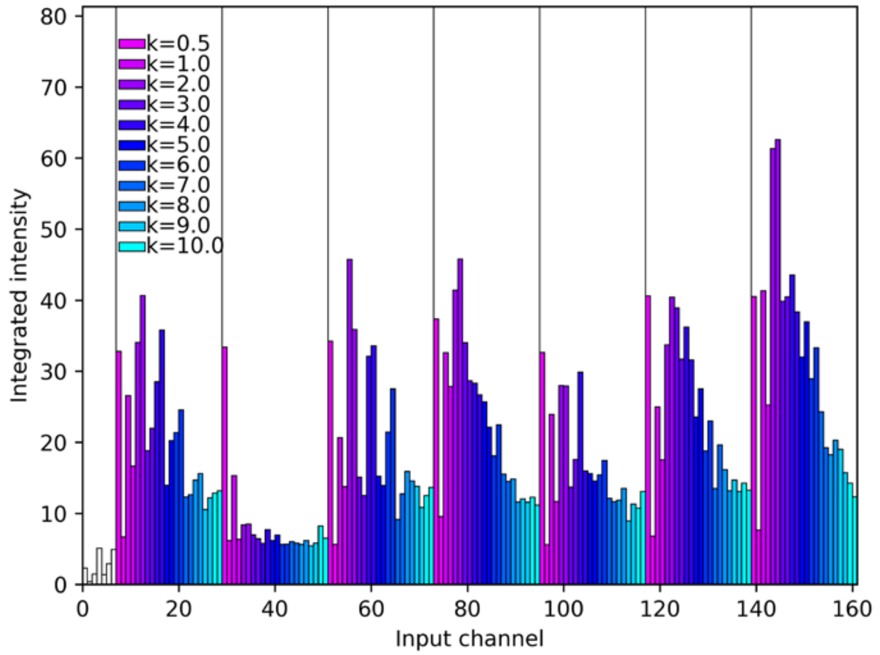

Figure S2. Analysis of the weights in the matrices connecting the input layer to the first hidden layer at the example of α-NPD. The first seven input channels are the dihedral angles α_i (white bars). The following channels are $\cos(k_j\alpha_i)$ and $\sin(k_j\alpha_i)$ terms for all periodicities k_j given in the legend and all angles α_i. The angles are defined in **Figure S3**.

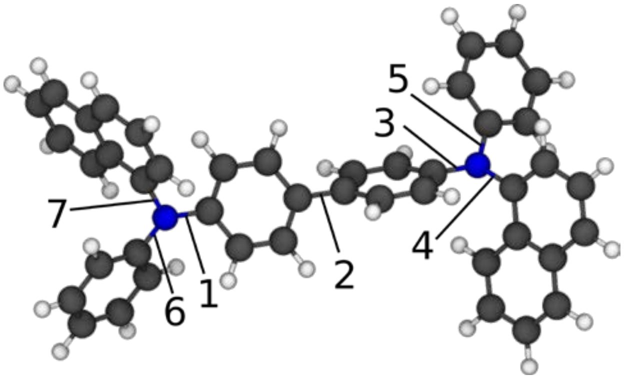

Figure S3. Definition of the dihedral angles shown in **Figure S2**.
